# Paroxysmal nocturnal hemoglobinuria in systemic lupus erythematosus: a case report

**DOI:** 10.1186/1752-1947-5-550

**Published:** 2011-11-14

**Authors:** Norio Nakamura, Toshiyuki Sugawara, Ken-ichi Shirato, Ryuichiro Kumasaka, Masayuki Nakamura, Michiko Shimada, Takeshi Fujita, Reiichi Murakami, Yuko Shimaya, Hiroshi Osawa, Hideaki Yamabe, Ken Okumura

**Affiliations:** 1 Community Medicine, Hirosaki University Graduate School of Medicine, 5 Zaifu-cho, Hirosaki-city, Aomori, 036-8562, Japan; 2 Department of Nephrology, Hirosaki University School of Medicine, 5 Zaifu-cho, Hirosaki-city, Aomori, 036-8562, Japan

## Abstract

**Introduction:**

Paroxysmal nocturnal hemoglobinuria is an acquired disorder of hemopoiesis and is characterized by recurrent episodes of intravascular hemolysis due to an increased sensitivity to complement-mediated hemolysis. Systemic lupus erythematosus with paroxysmal nocturnal hemoglobinuria is very rare. We report a case of paroxysmal nocturnal hemoglobinuria that developed in a patient with systemic lupus erythematosus and lupus nephritis.

**Case presentation:**

A 29-year-old Mongolian woman had systemic lupus erythematosus, which manifested only as skin lesions when she was 12 years old. She had leg edema and proteinuria when she was 23 years old, and a renal biopsy revealed lupus nephritis (World Health Organization type IV). She had been treated with steroids and immunosuppressant therapy. At 29, she had headaches, nausea, general fatigue, and severe pancytopenia and was admitted to our hospital. A laboratory evaluation showed hemolytic anemia. Further examination showed a neutrophil alkaline phosphatase score of 46 points, a CD55 value of 18%, and a CD59 value of 78.6%. The results of Ham test and sugar water tests were positive. The constellation of symptoms throughout the clinical course and the laboratory findings suggested paroxysmal nocturnal hemoglobinuria.

**Conclusions:**

To the best of our knowledge, systemic lupus erythematosus with paroxysmal nocturnal hemoglobinuria is very rare. Clinicians should be aware of the association between autoimmune and hematological diseases.

## Introduction

Paroxysmal nocturnal hemoglobinuria (PNH) is an acquired disorder of hemopoiesis and is characterized by recurrent episodes of intravascular hemolysis due to an increased sensitivity to complement-mediated hemolysis [1]. Systemic lupus erythematosus (SLE) with PNH is very rare. We present a case of PNH that developed in a 29-year-old woman who had SLE.

## Case presentation

A 29-year-old Mongolian woman had SLE, which manifested only as skin lesions when she was 12 years old. Because she had leg edema and proteinuria with serological and hematological abnormalities - the titers of anti-nuclear antibody and double-stranded DNA (dsDNA) antibody were increased and the lymphocyte count was decreased - at 23 years old, a renal biopsy was performed. The results revealed lupus nephritis (World Health Organization type IV). Her condition was diagnosed as SLE according to the criteria of the American College of Rheumatology [2]. She had been treated with steroids and immunosuppressants, including cyclophosphamide. She had pancytopenia at 25 years old, and secondary aplastic anemia, probably due to cyclophosphamide, was diagnosed. Pancytopenia was worsening six months after the onset of pancytopenia and therefore cyclosporine A was administered. When she was 28 years old, rheumatoid arthritis was diagnosed because of polyarthralgia and morning stiffness. After 2 months, she had severe headaches, and cerebral venous thrombosis was diagnosed by computed tomography. Laboratory data showed a high level of lactate dehydrogenase (LDH), a low level of haptoglobin, and a negative Coombs test result. These results suggested that she had hemolytic anemia, and the dose of steroid was increased. Her condition improved gradually.

**Figure 1:**
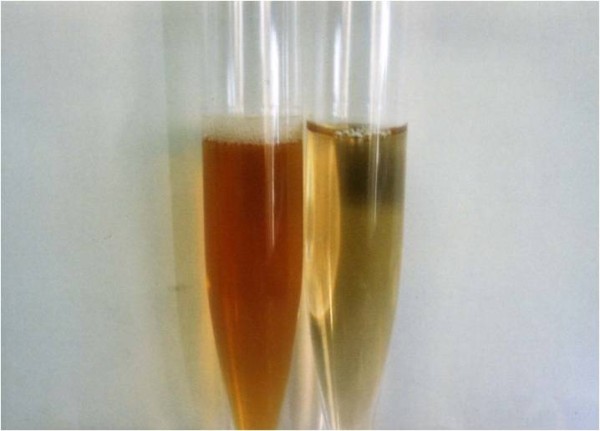
**Patient's urine (left) and control urine (right)**. The patient's urine was red because of hemolysis.

When she was 29 years old, she had headaches, nausea, general fatigue, and severe pancytopenia and was admitted to our hospital. A laboratory evaluation showed the following: hemoglobin of 7.3 g/dL, white blood cell count of 11,400/μL, platelets of 4.2 × 10^4^/μL, total protein of 4.9 g/dL, albumin of 2.3 g/dL, LDH of 1085 U/L, total bilirubin of 1.6 mg/dL, blood urea nitrogen of 34 mg/dL, creatinine of 1.1 mg/dL, C-reactive protein of 11.5 mg/dL, haptoglobin of less than 6 mg/dL, and dsDNA antibody of 5 IU/L. The results of direct and indirect Coombs tests were negative. Further examination showed a neutrophil alkaline phosphatase score of 46 points, a CD55 value of 18%, and a CD59 value of 78.6%. The results of Ham test and sugar water tests were positive. Her urine was red because of hemolysis (Figure 1). The constellation of symptoms throughout the clinical course and the laboratory findings suggested PNH.

## Discussion

PNH is an acquired disorder of hemopoiesis and is characterized by recurrent episodes of intravascular hemolysis due to an increased sensitivity to complement-mediated hemolysis [1]. A crucial pathophysiological mechanism is an acquired defect of the glycosylphosphatidylinositol-anchored proteins, namely CD55 and CD59 [3].

Flow cytometry analysis of red blood cells with monoclonal antibodies directed against CD55 and CD59 is now the gold standard technique for the diagnosis of PNH [4]. The normal values of CD55 and CD59 are more than 85.4% and more than 99.8%, respectively. In the present case, the values of CD55 and CD59 were 18% and 78.6%, respectively. Consequently, PNH was diagnosed.

PNH presents three clinical manifestations: (a) an acquired intravascular hemolytic anemia due to the increased susceptibility of the erythrocyte membrane to complement-mediated lysis; (b) thrombosis in large vessels, such as hepatic, abdominal, cerebral, and subdermal veins; and (c) mild to severe bone marrow hypoplasia that results in different degrees of pancytopenia. The triad of hemolytic anemia, thrombosis, and pancytopenia makes PNH a truly unusual clinical syndrome [5]. These manifestations were visible in our case.

Deficient expression of CD55 and CD59 has recently been reported in patients with autoimmune hemolytic anemia, autoimmune thrombocytopenia, or SLE [6,7]. An autoimmune condition such as SLE may contribute to the pathogenesis of PNH [8]. It is a very interesting phenomenon and might be associated with the pathogenesis of our present case.

## Conclusions

To the best of our knowledge, SLE with PNH is very rare and its mechanism is unknown. Clinicians should be aware of the association between autoimmune disease and PNH.

## Consent

Written informed consent was obtained from the patient for publication of this case report and any accompanying images. A copy of the written consent is available for review by the Editor-in-Chief of this journal.

## Abbreviations

dsDNA: double-stranded DNA; LDH: lactate dehydrogenase; PNH: paroxysmal nocturnal hemoglobinuria; SLE: systemic lupus erythematosus.

## Competing interests

The authors declare that they have no competing interests.

## Authors' contributions

NN wrote the manuscript and was a treating physician for the patient. TS, KS, RK, MN, MS, TF, and RM were also treating physicians for the patient. YS and HO performed the literature search and helped to write the manuscript. HY and KO were the major contributors to the writing of the manuscript. All authors read and approved the final manuscript.
